# Mechanosensitive Regulation of Fibrosis

**DOI:** 10.3390/cells10050994

**Published:** 2021-04-23

**Authors:** Shuying Yang, Sergey V. Plotnikov

**Affiliations:** Department of Cell & Systems Biology, University of Toronto, Toronto, ON M5S 3G5, Canada; rosiey.yang@mail.utoronto.ca

**Keywords:** fibrosis, extracellular matrix, durotaxis, tissue stiffness, inflammation, mechanosensing, mechanotransduction

## Abstract

Cells in the human body experience and integrate a wide variety of environmental cues. A growing interest in tissue mechanics in the past four decades has shown that the mechanical properties of tissue drive key biological processes and facilitate disease development. However, tissue stiffness is not only a potent behavioral cue, but also a product of cellular signaling activity. This review explores both roles of tissue stiffness in the context of inflammation and fibrosis, and the important molecular players driving such processes. During inflammation, proinflammatory cytokines upregulate tissue stiffness by increasing hydrostatic pressure, ECM deposition, and ECM remodeling. As the ECM stiffens, cells involved in the immune response employ intricate molecular sensors to probe and alter their mechanical environment, thereby facilitating immune cell recruitment and potentiating the fibrotic phenotype. This powerful feedforward loop raises numerous possibilities for drug development and warrants further investigation into the mechanisms specific to different fibrotic diseases.

## 1. Introduction

From the ancient times of Aristotle to the modern day, man’s understanding of mechanics has evolved greatly beyond basic physical laws deduced from the study of a lever [1]. Humans have since utilized the vast array of physical laws to build sophisticated machines, explore beyond the Earth’s atmosphere, and drastically transform our way of life. Contrary to our relatively recent discovery of mechanics, cells in the human body have long been utilizing the same physical principles to navigate complex microenvironments and operate microscopic machinery. However, this is not a one-way street—mechanical stimuli from their surroundings also affect cells, prompting them to push and pull, break down and build, and transport and retain, reshaping the very environment they reside in.

The growing body of work on mechanical properties of tissue in the past two decades shows growing appreciation for tissue stiffness—the resistance to deformation in response to applied force—in a myriad of biological processes. Spatial and temporal variations in tissue stiffness control gene expression and, ultimately, determine the differentiation lineages of stem cells [2]. Tissue stiffness also controls the activity of major intracellular signaling pathways and, through this mechanism, modulates cell proliferation, metabolic activity, and interactions of cells with their neighbors and the surrounding matrix [3,4]. Last but not least, long- and short-scale gradients in tissue stiffness act as potent and universal guidance cues directing migrating cells during developmental morphogenesis and cancer dissemination [5,6,7].

Furthermore, tissue stiffness has been shown to be involved in inflammation, a biological process long believed to be facilitated exclusively by biochemical factors. In this review, we highlight evidence supporting the critical role of stiffness in inflammation and fibrosis. In addition, we discuss key molecular mechanisms that cells employ to sense and respond to their mechanical microenvironment and how such mechanisms are intertwined with inflammatory pathways mediated by canonical soluble factors.

## 2. Tissue Stiffness—A Hallmark of Inflammation

Tissues in the human body naturally vary in stiffness. The stiffness of healthy tissue can range from hundreds of pascals (stiffness of panna cotta) in the brain to gigapascals in bone (stiffness of glass and steel) [8,9,10,11]. Other tissues, such as the breast and skeletal muscle, lie between the two extremes (800 Pa and 12 kPa, respectively) [12]. In a diseased state, however, some tissues display a marked increase in stiffness that can easily be detected by hand. Clinical practitioners often use palpation, the method of using one’s hands to examine the state of body organs, to detect such signs of disease.

**Figure 1:**
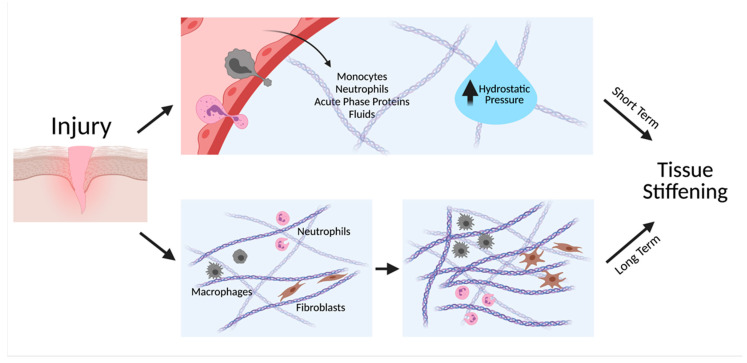
Inflammation facilitates both short-term and long-term tissue stiffening. Inflammatory cytokines induce vasodilation and an increase in permeability of vasculature, increasing leakage of fluids, proteins, and cells to the tissue, thereby increasing hydrostatic pressure. Long-term tissue stiffening occurs when immune cells and fibroblasts continuously deposit and remodel the ECM. Created with BioRender.com.

Inflammation-induced changes in tissue stiffness can be transient or long-term (Figure 1). During acute inflammation, resident macrophages and mast cells at the injured site sense harmful stimuli and release inflammatory mediators that quickly diffuse into the surrounding tissue. This triggers vasodilation, an increase in vascular permeability, and upregulation of adhesion molecules on leukocytes that together facilitate leukocyte extravasation and fluid leakage [13]. Such chemical signals also facilitate the migration as well as proliferation of leukocytes in the wound. Accumulation of fluids, acute phase proteins, neutrophils, and monocytes raises local hydrostatic pressure and causes visible swelling and redness of the tissue [14]. This short-lived upregulation of local tissue stiffness can be resolved within days as inflammation subsides. Chronic inflammation, however, often leads to long-term stiffening of tissue that is less reversible. Chronic inflammation is characterized by persistent inflammation in the absence of infectious agents, often attributed to a failure to resolve the acute inflammatory phase [15]. Repeated stimuli by inflammatory mediators prompt cells to rearrange their cytoskeleton, deposit ECM proteins, and remodel the local matrix, causing the tissue to stiffen up to 8-fold [16]. Diseases such as fibrosis and cancer are products of such tissue remodeling effects of chronic inflammation.

Increases in tissue stiffness are primary attributed to changes in the extracellular matrix (ECM), a scaffold composed of hundreds of proteins that connect stromal cells to maintain and support the structural integrity of tissues and organs. Main components of the ECM include collagen, fibronectin, and elastin. Collagen, the most abundant protein in the human body, is a heterotrimeric helix containing an abundance of glycine-proline-hydroxyproline repeats in the center and lysine residues at the ends. The helical structure of collagen molecules enables them to interact with each other laterally and form a staggered array of fibrils [17]. Post-translational modifications including oxidation, glycosylation, and hydroxylation crosslink collagen, further increasing the stiffness of individual collagen fibrils to the megapascal range [18,19]. Among the 28 collagens identified in vertebrates, collagens I, II, III, V, and XI are the primary fibril-forming collagens, with collagen I being the most prevalent in fibrotic lesions [20]. Other collagens including collagen IV, which form meshlike networks due to disruptions in the amino acid repeats, are not directly involved in tissue stiffening during fibrosis and will not be discussed in this review.

The key cell type that deposits collagen I during chronic inflammation is fibroblasts. Unlike the leukocytes that precede them at the onset of inflammation, these cells are activated later in the process and heavily depend on their adherence to the underlying ECM. In healthy tissue, fibroblasts provide maintenance to their surroundings through controlled collagen deposition and degradation; however, inflammatory mediators secreted by a number of immune cells may upregulate their collagen synthesis. Macrophages, among others, secrete cytokines including IL-1, IL-4, IL-13, TNFα, and TGFβ upon activation [21,22]. The complex mechanism of macrophage activation has been thoroughly reviewed in a number of papers [23,24]. Binding of IL-4 and IL-13 to their corresponding receptors on fibroblasts promotes the transcription of collagen I genes through STAT6, a transcription factor that shuttles from the cytoplasm to the nucleus upon tyrosine phosphorylation by the receptors [25,26,27,28]. Such signaling events have also been reported to occur through the JNK and ERK pathways [29,30]. IL-1 and TNFα signal through NF-κB to upregulate the expression of collagen-producing cytokines TGFβ and PDGF that simultaneously recruit more immune cells via chemotaxis [31,32,33]. Interestingly, macrophage-derived IL-10 inhibits collagen synthesis, suggesting a regulatory role of cytokines in ECM deposition [34,35].

An elegant study conducted by the Riley lab recently revealed a striking new role of macrophages in collagen production—in addition to indirect signaling pathways, macrophages directly deposit collagen during scar formation in the heart [36]. The group found elevated levels of collagen and related ECM genes expressed in macrophages in infarcted hearts of both zebrafish and mice. Although macrophages were present in the same location one day after cardiac injury, the *col1a2* gene was only expressed in the infarcted zone seven days post injury. Transplantation of such macrophages into wild type mice induced cardiac scar formation one week after surgery, further supporting the role of inflammation in ECM deposition.

Among the multitude of proinflammatory growth factors secreted by immune cells and resident fibroblasts, TGFβ is arguably one of the heaviest studied. This legendary growth factor has been implicated in numerous biological processes, including development, cell migration, and a myriad of diseases [37,38]. Like many other cytokines, TGFβ is synthesized in its prohormone state, attached to a signal sequence and an *N*-terminal latency-associated peptide (LAP). Upon cleavage, the active segment remains encapsulated by LAP and binds latent TGFβ binding proteins [39]. The complex, known as the large latent complex or LLC, is secreted to the extracellular space and needs to be unraveled before TGFβ can bind its receptors. Binding-induced dimerization of the receptor tyrosine kinases promote phosphorylation of downstream SMAD proteins that translocate to the nucleus and facilitate transcription of target genes including collagen and alpha smooth muscle actin (αSMA) [40]. In the immune response, TGFβ is synthesized by platelets, macrophages, and fibroblasts, and is key to the transdifferentiation of fibroblasts into another collagen-producing cell type, myofibroblasts.

Myofibroblasts are highly contractile cells responsible for the closure of open wounds. Characterized by increased levels of αSMA and pronounced actin bundles, these cells exert traction forces two to three times higher than their precursor fibroblasts [41], although recent studies indicate that other cell types such as endothelial cells and fibrocytes can also give rise to myofibroblasts [42]. In addition to collagen production [43], myofibroblasts are known for their ability to remodel the matrix through contractile forces. High levels of pulling force align individual collagen bundles parallel to the direction of force, providing a paved path for immune cell, fibroblast, and myofibroblast recruitment to the wounded site [44,45,46]. While thin, short collagen segments are barely visible under a second harmonic generation microscope, the accumulation of aligned fibrillar collagen manifests as thick, interconnected bundles that are distinct from their counterparts. Such increases in fiber alignment not only change the architecture of the collagen scaffold but also increase its local stiffness [47]. It is thus not surprising that dysregulated, sustained activation of myofibroblasts and fibroblasts lead to large-scale tissue remodeling that alter the mechanical properties and function of the organ.

In response to increased collagen deposition, cells have evolved a defense mechanism to resolve the scar tissue produced. Neutrophils, macrophages, fibroblasts, and myofibroblasts secrete matrix metalloproteinases (MMPs)—zinc-dependent endopeptidases that degrade the ECM—to cleave matrix proteins such as collagen and fibronectin (FN). MMPs upregulated during the immune response and in fibrotic tissues include MMP1-3, -7, and -9, among others [48,49]. Although the 23 human MMPs are structurally similar, they have been shown to play nonredundant roles and exhibit tissue-specific functions [50]. One well studied example is MMP-1, also known as collagenase I. While MMP-1 facilitates directed keratinocyte migration to restore damaged epithelium [51], it upregulates the expression of the VEGF receptor in endothelial cells during vascular remodeling [52], suggesting a nonlinear role of MMPs in wound healing and fibrotic processes. In fact, many MMPs have been found to exhibit tissue-specific profibrotic signaling activity, the details of which are discussed in several reviews [50,53]. Briefly, the signaling activity of MMPs arises from their ability to fragment ECM proteins into small segments containing domains that activate integrins on resident cells, inducing collagen deposition and fibroblast-to-myofibroblast transdifferentiation, both potent drivers of fibrosis. ECM cleavage-independent functions of MMPs include cleaving extracellular domains of growth factor receptors and releasing TGFβ from the LLC [54]. In light of these findings, much effort has been devoted to the development of MMP inhibitors as therapeutics for fibrosis [49,55]. Moreover, the remodeled ECM can be biochemically distinct from the original ECM, containing pronounced levels of lysyl oxidase (LOX). LOX is a copper metalloenzyme that covalently crosslinks collagen through oxidation of its lysine and hydrolysine residues outside of the triple helical domain [56]. Early observations showed that the combined stimulation with growth factors FGF and IGF-1 increased *lox* gene expression in the oral cavity of the rat, especially near inflamed lesions [57]. Although the available techniques did not allow for precise determination of the cell type of interest, the authors deduced from their localization and morphology that the *lox*-expressing cells were fibroblasts. These data suggest that tissue remodeling by MMPs may not resolve scar tissue but instead, replace healthy tissue with stiffer ones.

## 3. Tissue Stiffness—A Driver of Inflammation

As soluble cues prompt the microenvironment to stiffen, the remodeled matrix is not only a product, but also a driver of inflammation. Mechanical cues have been shown to play principal roles in immune cell migration, the formation of immunological synapses, and the exacerbation of chronic inflammatory diseases including fibrosis and cancer [58,59,60]. Such cellular responses to tissue stiffness require the cells to probe and survey the mechanical properties of the microenvironment through rigidity mechanosensing. This section provides a comprehensive review of the mechanosensing mechanisms.

### 3.1. Molecular Sensors of Stiffness

**Figure 2:**
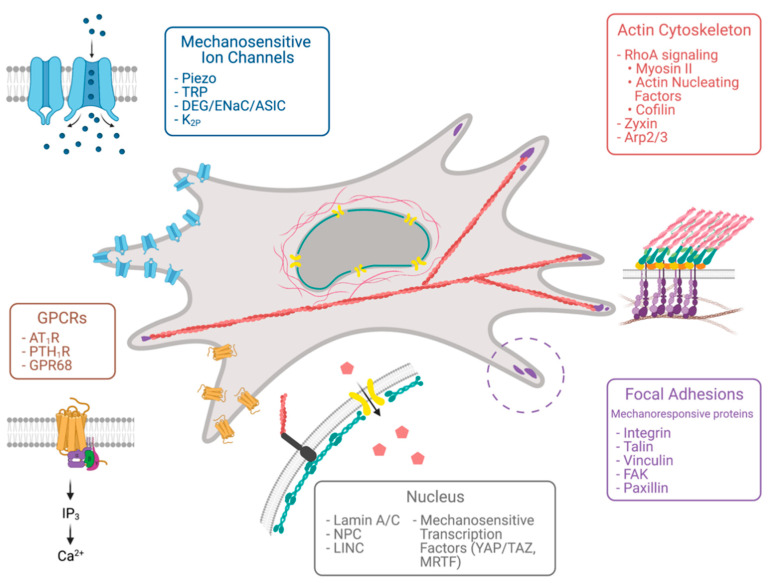
Molecular sensors of tissue stiffness. Various cellular compartments respond to mechanical force, including focal adhesion proteins, the actin cytoskeleton, the nucleus, mechanosensitive ion channels, and G-protein-coupled receptors. Created with BioRender.com.

Cells employ a variety of mechanisms to probe the mechanical property of their surroundings. Despite involving different players, such mechanisms all evolve around force-sensitive proteins that transduce extracellular mechanical stimuli to intracellular chemical signals that ultimately affect a cell’s decision (Figure 2).

#### 3.1.1. Focal Adhesions/Focal Contacts

At the center of mechanosensitive proteins lies the protein complex focal adhesion, integrin-dependent anchorages linking the ECM to the cell’s actin cytoskeleton. With over 180 associated proteins identified up to date [61], focal adhesions are arguably one of the most complex protein structures in the cell and are important for cell proliferation, cell shape, and migration. The myriad of proteins assembles into several functional layers in the focal adhesion, including a layer of transmembrane integrins that directly bind to the ECM, a membrane-proximal integrin signaling layer, a force transduction layer, and an actin regulatory layer that is bound to filamentous actin [62]. Force generated within the cell and from the microenvironment propagates through these layers bidirectionally, allowing the cell to both actively probe and passively sense the mechanical properties of the ECM. Integrins in the bottom-most layer are transmembrane α and β heterodimers that bind RGD-domain-containing ECM proteins such as collagen and fibronectin. The inactive integrin exists in a folded state and undergoes both outside-in activation by ECM binding and inside-out activation by talin, a key force-transducing adaptor protein that binds β integrin cytoplastic tails on one end and filamentous actin on the other. Following integrin activation, intracellular adaptors and signaling proteins are recruited to the integrin signaling layer. Mechanical stimulation has been shown to affect two main inhabitants of this layer, focal adhesion kinase and paxillin, by upregulating the amount and activity of focal adhesion kinase and increasing phosphorylation of paxillin [63,64,65]. Talin further extends into the force transduction layer, where cryptic binding sites for the actin-binding protein vinculin unfolds upon force, further reinforcing the connection between the matrix and the actin cytoskeleton [66,67,68]. Together, these proteins form the skeleton of the molecular “clutch” that mediates cell–ECM adhesion and mechanotransduction.

#### 3.1.2. Actin Cytoskeleton

Intracellular forces transmitted to focal adhesions are generated by the dynamic actin cytoskeleton, another key contributor to cellular mechanosensitivity. Myosin II, contractile motors that pull on antiparallel actin filaments, drives traction forces that enable cells to sample the rigidity of the ECM. Such contractile forces are upregulated when the small GTPase RhoA activates its downstream effector ROCK. ROCK further upregulates phosphorylation of the myosin light chain by both direct phosphorylation and inhibition of myosin light chain phosphatase [69]. While early research showed that cells exert stronger traction forces on stiff substrates than on soft substrates [70], recent studies found that this force is not necessarily stable [71]. A subset of focal adhesions may exert fluctuating traction forces on the ECM, analogous to humans repeatedly tugging on a surface by hand. This force transmitted through the focal adhesion kinase/phospho-paxillin/vinculin axis is critical for the cell to tug at a wide range of substrate rigidities and acquire information from their microenvironment, which in turn affects contractility and allows cells to migrate towards stiffer substrates [72]. In addition, contractile forces increase the tension that focal adhesions can sustain by strengthening cell–ECM adhesions and prolonging the lifetime of these bonds (termed “catch bonds”). As mentioned above, force applied on talin unfolds cryptic vinculin binding sites that reinforce the cell–ECM linkage [66]. Contractility also induces conformational changes on fibronectin molecules that expose buried synergy sites, further activating integrins and recruiting intracellular focal adhesion proteins [73,74]. Inhibition of myosin-II-mediated contractility, on the contrary, results in diffuse integrin distribution and a lack of focal adhesion in cells [75]. Actin polymerization independent of myosin II activity has also been implicated in cell mechanosensing. Depletion of the actin elongation factor Dia1 decreases the magnitude of traction force exerted on the ECM compared to control cells, potentially limiting the range of stiffness sensed by adhesions [76,77]. Similarly, inhibition of the actin nucleation factors of the Ena/VASP family perturbs stiffness-dependent cell spreading and the ability of cells to durotax in three-dimensional microenvironment [78].

#### 3.1.3. Nucleus

As the hub of genetic material, the mammalian cell nucleus has long been perceived as a hub of biochemical reactions, with its mechanical properties overlooked. Yet, an increasing body of evidence suggests that in addition to its striking stiffness, it is one of the most important mechanosensitive structures in the cell [79]. Indeed, while enucleated cells retain their ability to polarize and migrate on a planar two-dimensional substrate, optimal migration velocity is only attained at higher stiffness (8.6 kPa and 25 kPa, respectively), suggesting a role of nucleus-mediated mechanosensitivity in cell migration [80]. Such mechanosensitivity is conferred by lamin A-mediated contractility and the LINC complex that connects the nuclear lamina to cytoplasmic actin. In three-dimensional environments, the nucleus takes on an additional role to maintain its mechanical integrity as cells navigate through stiff, dense matrices that exert forces and deformations at a significantly larger scale. Proteomic analysis by the Discher group show that the amount of lamin A positively correlates with an increase in collagen-dependent tissue stiffness, thereby maintaining the structural integrity of the nucleus when exposed to compressive forces in tissues [81]. Interestingly, upregulation of protective lamin A also facilitates osteogenesis while suppressing adipogenesis, implicating the mechanosensitivity of the nucleus in gene expression. Such changes in gene expression have been attributed to the nuclear translocation of force-sensitive transcription factors MRTF, YAP, and TAZ, possibly through the opening of nuclear pores [82,83,84]. For more details on the mechanical regulation of transcription, we refer readers to review articles [85]. Recently, the Piel group suggested a model in which the nuclear membrane expands and stretches upon confinement, changing the conformation of stretch-activated channels to release calcium and other signaling molecules that promote actomyosin contractility in the cytoplasm [86]. Such lamin-A-dependent mechanosensitivity not only affects HeLa cells but also promotes dendritic cell migration under confinement. As the Piel group elegantly describes, the nucleus acts as a ruler that probes its physical surroundings, resulting in changes in its structural and biochemical composition that facilitate downstream signaling.

#### 3.1.4. Mechanosensitive Ion Channels

Stretch-activated channels exist not only in the nucleus but also on the plasma membrane. Well-known examples of such channels include Piezo channels, transient receptor potential (TRP) channels, and a subset of sodium and potassium ion channels (Figure 2). Extensive studies on the mechanism of their gating have revealed two possible models—the lipid force model and the tether force model. The underlying assumption of the lipid force model is that the lipid bilayer encapsulating any cell is inherently anisotropic, meaning that although the plasma membrane is only 5 to 10 nm in thickness (the lipid bilayer being even thinner), intrinsic forces differ greatly at various depths of the bilayer [87]. In addition, the geometry of the bilayer can be greatly altered depending on the local composition due to differences in lipid shape. Phospholipids that resemble cylinders assemble into bilayers while lysophospholipids that are considered conical lipids preferentially form micelles [88]. Any alteration in curvature resulting from local composition and force profile changes may allow transmembrane ion channels to undergo conformation changes to open or close. The lipid force model thus postulates that lipids alone can gate mechanosensitive ion channels [89]. The tether force model, on the other hand, attributes changes in channel conformation to proteins capable of tethering the channel to structures on either side of the plasma membrane. Examples of such tethers include the actin cytoskeleton, adaptor proteins that bind to actin, and extracellular scaffolds. While TRP members such as TRPC6 bind to actin indirectly through PDZ-domain-containing adaptor proteins, TRPV2 and TRPV4 have been shown to interact directly with actin [90,91,92]. It is thus not surprising that forces generated through myosin contractility and actin flow may propagate to such ion channels and facilitate channel opening. An example of extracellular tether utilization can be found within the stereocilia of hair cells in the inner ear. Tip links connect stereocilia to their neighbors and are bound to mechanosensitive channels that transmit auditory information. When stereocilia is collectively deflected by force, tension in the tip links increases as stereocilia exit their resting position [93]. After the initial deflection, tension decreases and allows the subsequent closure of the channel.

#### 3.1.5. G-Protein-Coupled Receptors

In addition to ion channels gated by mechanical force, G-protein-coupled receptors (GPCRs) including angiotensin II type 1, parathyroid hormone receptor type 1, and the endothelin ET1A receptor have emerged as an important class of mechanosensitive proteins important for biological processes in the vasculature and heart [94,95,96]. The angiotensin II type 1 receptor was the first GPCR found to be sensitive to mechanical force [94,96]. In their seminal study, Zou et al. showed that cardiomyocytes subject to mechanical stretch exhibited angiotensin-II-independent upregulation of ERK, a downstream effector of the angiotensin II signaling pathway [94]. In addition, treating mice with the angiotensin II type 1 receptor inhibitor reduced the development of mechanical-load-induced cardiac hypertrophy. Moreover, a high-throughput mechanical stimulation assay recently revealed that GPR68, a proton-activated GPCR involved in pH homeostasis, inflammation and fibrosis, responds to mechanical stimulation [97,98,99,100]. Specifically, laminar sheer forces induced Ca^2+^ transients in endothelial cells residing in relatively thin arteries. Although the mechanism through which such GPCRs confer mechanosensitivity is unclear, several groups have attributed this activity to conformational change [101,102]. FRET data obtained by Erdogmus et al. showed that conformational changes in helix 8 induced signaling events downstream of Gq/11-, Gi/o-, and Gs-protein-coupled receptors, shedding light on mechanisms that aid drug development [102].

### 3.2. Inflammatory and Mechanosensitive Pathways Intertwined

**Figure 3:**
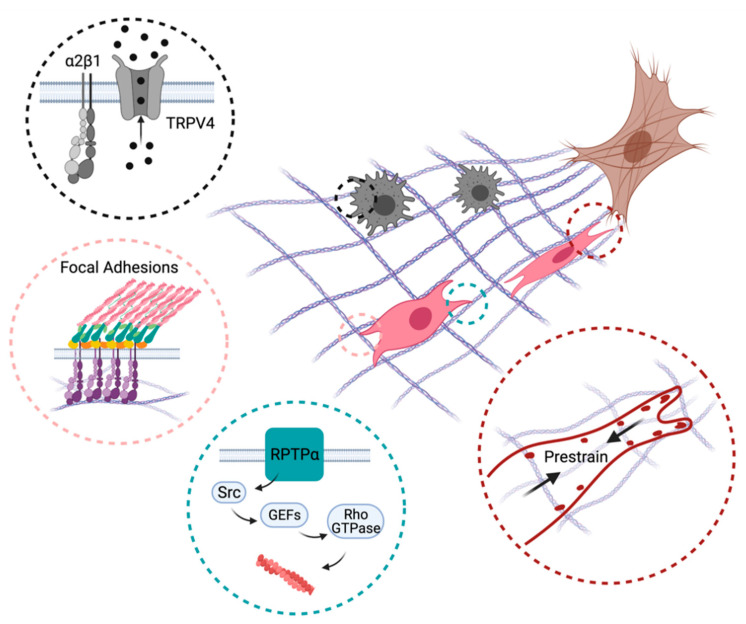
Mechanisms promoting directed immune cell and fibroblast migration towards inflammatory lesions (brown). Myofibroblast-induced collagen remodeling creates a gradient of ECM stiffness and alignment that recruits macrophages (black) and fibroblasts (pink) to the wound site. Black circle: α2β1 integrins and mechano-gated ion channels such as TRPV4 enable macrophages to sense ECM deformation fields produced by contractile myofibroblasts. Pink circle: Mechanical interactions mediated by dynamics of integrin-based focal adhesions allow fibroblasts to migrate up the ECM stiffness gradient. Green circle: Signaling proteins (RPTP-α) not directly involved in cell–ECM adhesion regulate directed migration through the actin cytoskeleton and cellular protrusions. Red circle: Fibroblasts prestrain collagen fibers through myosin contractility and adhesions to enable directed migration. Created with BioRender.com.

Despite being loyal employers of chemical signals, immune cells and their effectors also utilize the molecular sensors of stiffness detailed above in response to injury (Figure 3). The chronic inflammatory disease fibrosis is a classic example in which mechanosensitive pathways respond to the stiffened ECM and exacerbate inflammation, contributing to a feedforward loop that potentiates tissue injury and organ dysfunction.

Recruitment of immune cells and adherent mesenchymal cells (fibroblasts and myofibroblasts) through durotaxis, directional migration from softer to stiffer regions of the ECM, has been proposed to be a key factor of fibrosis [103]. In a physiological context, ECM stiffness gradients have been observed in numerous fibrotic diseases including idiopathic pulmonary fibrosis, liver cirrhosis, and lung fibrosis [104]. Such increase in tissue stiffness at the fibrotic lesion is often accompanied by altered ECM composition, which can further facilitate directed cell migration [105].

Experimental data provide the mechanism through which the rigid tissue matrix may attract migrating cells. The mechanism of durotaxis may be entirely mechanical and explicable in terms of how strong a protruding cell edge can push or pull on the ECM without losing energy for deforming the substrate. On an ECM with a gradient of stiffness, the cell edge attached to the stiffer substrate dissipates less energy and advances faster compared to its counterpart on the softer substrate, and such a difference in cell edge dynamics is sufficient to translocate the entire cell up an ECM stiffness gradient. This mechanism, first proposed by Harland et al., is supported by mathematical modeling as well as by several lines of experimental evidence [106]. Analysis of cell migration upon depletion of vinculin has demonstrated that fibroblasts with impaired attachment to the ECM are unable to durotax [107]. The importance of cell attachment to the ECM via integrin-based focal adhesions was further supported by the observation that depletion of myosin II, a master regulator of focal adhesion assembly, abolishes durotaxis [108]. Suppression of focal adhesion disassembly by inhibiting focal adhesion kinase was shown to perturb durotaxis [109]. Together, these data demonstrate that dynamic mechanical interactions between the cytoskeleton and the substrate on which the cell is moving is a prerequisite for durotaxis.

Recently, this simple mechanical model of durotaxis has been challenged by the discovery of signaling proteins, which do not regulate cytoskeleton dynamics and cell–ECM interaction directly but are essential for durotaxis. The receptor-type protein tyrosine phosphatase alpha (RPTP-α) was the first regulatory protein found to be essential for a variety of mechanosensitive cellular responses, including durotaxis [110]. This phosphatase, localized on the cell plasma membrane, transmits the mechanical signals from the cell microenvironment to Src family kinases. The activated kinases, in turn, phosphorylate tyrosine residues on an unknown Rac1/Cdc42 GTP exchange factor(s) resulting in activation of Rho GTPases, actin cytoskeleton reorganization, and cell edge protrusion [111]. Following this seminal discovery, the Turner group identified several protein regulators of Rho GTPases that are essential for durotaxis [112]. Recently, the mechanosensitive transcriptional regulator YAP was shown to be essential for cellular response to ECM stiffness gradients [113], highlighting the importance of cellular signaling for durotaxis.

Although the contribution of durotaxis to fibrosis has not been assessed directly, several lines of evidence indicate that mechanical gradients in tissue microenvironment recruit both immune and tissue-resident parenchymal cells to fibrotic lesions. Analysis of hepatic stellate cell migration on synthetic hydrogels mirroring rigidity gradients found within fibrotic pancreas revealed a strong bias in the direction of cell movement toward stiffer, fibrotic like regions of the substrates [114]. Suppression of focal adhesion kinase and myosin II, two proteins that are known to be essential for durotaxis, completely abrogated such directional migration, highlighting the conservation mechanisms of durotaxis across various cell types. Intriguingly, this study demonstrated a drastic increase in the speed of cells migrating along the stiffness gradient compared to their counterparts residing within stiff or soft areas of the substrate, reinforcing the central role of ECM mechanics in the recruitment of migratory cells to fibrotic lesions. Similarly, the distribution of mast cells in skin was shown to correlate with local ECM stiffness with a higher cell density observed at the boundary of stiff and soft tissues [115]. The mechanisms of such cell positioning within fibrotic tissue were recently investigated by the Campagnola group. By tracking individual cells migrating on microfabricated three-dimensional collagen scaffolds, Tisler et al. demonstrated that biomechanical features of collagen stroma affected by idiopathic pulmonary fibrosis greatly enhance cell polarity and facilitate directional cell movement along stiff and aligned collagen bundles [116]. It is tempting to speculate that contractile forces generated by myofibroblasts or other cell types in response to proinflammatory cytokines prestress and stiffen collagen fibers, and such mechanical gradients quickly propagate through tissue, attracting single cells and cell clusters to the fibrotic lesions [117,118,119].

Stiffness of the ECM is not the sole determinant of directed migration in immune response and fibrosis. Dynamic pulling forces generated by contractile myofibroblasts have been recently shown to recruit macrophages in three-dimensional collagen matrices [46]. Such directed migration toward mechanically active cells is likely independent of stiffness and alignment of the ECM but requires sensing of the directional pulling force propagated through fibrillar collagen. Mechanical activation of macrophages’ sensory system mediated by α2β1 integrins and mechano-gated ion channels can trigger a wide range of intracellular signaling pathways and ultimately lead to biased protrusive activity and directional migration. For example, upregulation of integrin-bound stretch-activated calcium channel TRPV4 was previously shown to facilitate cell migration by activating Cdc42/N-WASP signaling axis [120,121]. Recruitment of TRPV4 to focal adhesions in a force-dependent manner followed by local activation of small molecule GTPase Rac1 is an alternative mechanism to enhance protrusive activity by collagen contractions [122]. Similar to activation of Cdc42/N-WASP, an increase in Rac1 activity leads to an assembly of branched actin network that pushed on the plasma membrane driving the cell forward [123]. Furthermore, Rac1 was also shown to modulate the signaling state of focal adhesions and enhance cell migration by promoting rapid focal adhesion assembly [124]. Although the exact signaling events activated by periodic contractions of myofibroblasts remain to be elucidated, these studies accentuate the importance and complexity of mechanical interactions between the inhabitants of a fibrotic lesion and the need for further studies to identify the mechanisms that orchestrate mechanobiological responses in vivo.

Cooperation between cellular players exist beyond the mechanical level. Integrins, often emphasized for their role in mechanotransduction, have been shown to cooperate with chemical signaling axes mediated by growth factors, proinflammatory cytokines, and their downstream effectors to promote fibrosis.

Integrins cooperate with TGFβ in both a direct and indirect manner. TGFβ-induced integrin upregulation was first shown more than 25 years ago, where Zambruno et al. found an increased presence of α_5_β_1_, α_v_β_5_, and α_2_β_1_ integrins during keratinocyte-mediated wound healing [125]. Such upregulation of α_v_ integrins in myofibroblasts facilitate the release of TGFβ from the latency-associated peptide through α_v_β_6_-mediated force transduction, completing a feedforward loop in which the growth factor potentiates its own synthesis [126]. In addition, integrins and TGFβ signaling share a multitude of downstream effectors including MAPK, focal adhesion kinase, and Rho GTPases, all of which contribute to rearrangement of the actin cytoskeleton [127,128]. Such convergence of signaling cascades results in elevated levels of cellular contractility and pronounced stress fibers that are characteristics of fibrosis. Interestingly, integrins also exhibit antifibrotic roles, as shown by Pozzi et al. [129]. In this elegant study, Pozzi et al. found that the collagen-binding integrin α_1_β_1_ facilitated dephosphorylation of the TGFβ type II receptor intracellular tail through recruitment of the phosphatase TCPTP in kidney epithelial cells. Knocking out the α_1_ integrin facilitated EMT, as cells gained a fibroblast-like phenotype and displayed a marked increase in SMAD activity.

Other growth factors involved in fibrosis that synergize with integrins include EGF, PDGF, and VEGF, all of which are target nodes in antifibrotic drug development [130,131,132]. Specifically, β_1_ integrins stimulate the phosphorylation of EGFR in the absence of EGF, while simultaneously regulating EGF signaling by controlling EGFR endocytosis [133,134]. Unlike EGFR, VEGFR directly binds to α_9_β_1_ and α_v_β_3_ integrins in endothelial cells to promote angiogenesis, although the exact mechanism has yet to be elucidated [135,136]. Studies on the cooperation between such growth factor receptors and integrins in the context of fibrosis is lacking, but it is not beyond reason to speculate that clustering and upregulation of integrin expression in stiff, remodeled ECM has the potential to exacerbate profibrotic growth factor signaling.

## 4. Concluding Remarks

As mechanical cues slowly migrate towards the center of the cell biology stage, tissue stiffness has undoubtedly gained a significant amount of fame and recognition amongst the scientific community. Whether it be the inflammation exposition or the rising action of ECM stiffening, immune cells, mesenchymal cells, and all other actors in this theatrical piece acknowledge that they are of no significance without the presence of each other. In fact, the interplay between inflammatory and mechanosensitive players intensifies the climax of this theatrical piece and continues to deliver an ever more mesmerizing plot that has anything but an ending.
